# Combined FET PET/MRI radiomics differentiates radiation injury from recurrent brain metastasis

**DOI:** 10.1016/j.nicl.2018.08.024

**Published:** 2018-08-19

**Authors:** Philipp Lohmann, Martin Kocher, Garry Ceccon, Elena K. Bauer, Gabriele Stoffels, Shivakumar Viswanathan, Maximilian I. Ruge, Bernd Neumaier, Nadim J. Shah, Gereon R. Fink, Karl-Josef Langen, Norbert Galldiks

**Affiliations:** a Inst. of Neuroscience and Medicine (INM-3, -4, -5), Forschungszentrum Juelich, Juelich, Germany; b Dept. of Stereotaxy and Functional Neurosurgery, University of Cologne, Cologne, Germany; c Dept. of Neurology, University of Cologne, Cologne, Germany; d Dept. of Neurology, University Hospital RWTH Aachen, Aachen, Germany; e Dept. of Nuclear Medicine, University Hospital RWTH Aachen, Aachen, Germany; f Center of Integrated Oncology (CIO), Universities of Cologne and Bonn, Cologne, Germany

**Keywords:** Radiosurgery, Textural feature analysis, Radiation necrosis, Radiation-induced changes, FET PET

## Abstract

**Background:**

The aim of this study was to investigate the potential of combined textural feature analysis of contrast-enhanced MRI (CE-MRI) and static O-(2-[^18^F]fluoroethyl)-L-tyrosine (FET) PET for the differentiation between local recurrent brain metastasis and radiation injury since CE-MRI often remains inconclusive.

**Methods:**

Fifty-two patients with new or progressive contrast-enhancing brain lesions on MRI after radiotherapy (predominantly stereotactic radiosurgery) of brain metastases were additionally investigated using FET PET. Based on histology (*n* = 19) or clinicoradiological follow-up (*n* = 33), local recurrent brain metastases were diagnosed in 21 patients (40%) and radiation injury in 31 patients (60%). Forty-two textural features were calculated on both unfiltered and filtered CE-MRI and summed FET PET images (20–40 min p.i.), using the software LIFEx. After feature selection, logistic regression models using a maximum of five features to avoid overfitting were calculated for each imaging modality separately and for the combined FET PET/MRI features. The resulting models were validated using cross-validation. Diagnostic accuracies were calculated for each imaging modality separately as well as for the combined model.

**Results:**

For the differentiation between radiation injury and recurrence of brain metastasis, textural features extracted from CE-MRI had a diagnostic accuracy of 81% (sensitivity, 67%; specificity, 90%). FET PET textural features revealed a slightly higher diagnostic accuracy of 83% (sensitivity, 88%; specificity, 75%). However, the highest diagnostic accuracy was obtained when combining CE-MRI and FET PET features (accuracy, 89%; sensitivity, 85%; specificity, 96%).

**Conclusions:**

Our findings suggest that combined FET PET/CE-MRI radiomics using textural feature analysis offers a great potential to contribute significantly to the management of patients with brain metastases.

## Highlights

• Differentiation between brain metastasis recurrence and radiation injury is of high clinical importance.
• Differentiation based on contrast-enhanced conventional MRI is often inconclusive.
• Radiomics and hybrid amino acid PET/MR imaging are increasingly gaining attention in Neuro-Oncology.
• We investigated the potential of combined PET/MRI radiomics analysis using MRI and FET PET in patients with brain metastases.
• Combined PET/MRI radiomics allows the differentiation of brain metastasis recurrence from radiation injury with high accuracy.

## 1 Introduction

Over recent years, the treatment of brain metastasis using radiotherapy has evolved substantially. Treatment options include local postoperative external fractionated radiotherapy, stereotactic radiosurgery, interstitial brachytherapy, and whole-brain radiotherapy (Arvold et al., 2016). All these methods, applied solely or in combination, may lead to local radiation doses that exceed tolerance levels of normal brain tissue and may accordingly result in a radiation injury. The frequency of which radiation injury occurs depends on the applied method of radiotherapy. After stereotactic radiosurgery, radiation injuries occur in 5–34% of cases (Kohutek et al., 2015; Brown et al., 2017; Kocher et al., 2011; Sneed et al., 2015; Schüttrumpf et al., 2014). In contrast, a radiation injury is uncommon after fractionated (local or whole-brain) radiotherapy alone (incidence, 0–5%) (Andrews et al., 2004; Oehlke et al., 2015; Shin et al., 2015; Connolly et al., 2013) and is also rare after low-dose brachytherapy (Ruge et al., 2011). Typically, after radiosurgery, radiation injuries develop within a median time range of 7–11 months (Kohutek et al., 2015; Brown et al., 2017; Kocher et al., 2011; Sneed et al., 2015; Schüttrumpf et al., 2014). Moreover, in a subgroup of long-term survivors radiation injuries after stereotactic radiosurgery have also been reported after >5 years (median time, 33 months) (Fujimoto et al., 2018). Unfortunately, neurological signs and symptoms caused by both recurrent brain metastasis and radiation injury are indistinguishable and hence often impose severe clinical problems during the follow-up (Chao et al., 2013).

Contrast-enhanced MRI (CE-MRI) is routinely used in clinical practice for the follow-up of patients with previously irradiated brain metastases. However, recurrent brain metastases cannot easily be differentiated from radiation injury using conventional MRI (Stockham et al., 2012). More recently, the differentiation between radiation injury and recurrence of brain metastasis has been markedly improved by imaging parameters derived from static and dynamic amino acid PET scans, suggesting that a diagnostic accuracy in the range between 80 and 90% can be obtained (Ceccon et al., 2017; Cicone et al., 2015; Galldiks et al., 2012; Kickingereder et al., 2013; Lizarraga et al., 2014; Terakawa et al., 2008; Tsuyuguchi et al., 2003). However, dynamic FET PET scans require a more costly and time-consuming acquisition, data reconstruction and analysis and, thus, are not yet implemented in clinical routine.

Textural feature analysis of inconclusive lesions on PET (Lohmann et al., 2017) and MR images (Larroza et al., 2015; Nardone et al., 2016; Pallavi et al., 2014; Tiwari et al., 2016) is another promising approach. It is based on the assumption that the microstructure of a process depends on the underlying pathology and is reflected in subtle differences in the radiological image that cannot be detected by means of human perception but can be made accessible by high-dimensional quantitative image analysis often referred to as “radiomics”. It seems likely that such an approach could be improved by combining PET and MR image analysis, which may derive special features based upon complementary tissue properties.

Here, we report for the first time the usefulness of combined PET/MRI radiomics analysis using CE-MRI and FET PET scans in patients with brain metastases. Following simple normalization, reslicing, and resampling procedures of already obtained neuroimages, we demonstrate that freely available radiomics image analysis tools can be used to differentiate brain metastasis recurrence from radiation injury with a high accuracy, particularly when the information from both CE-MRI and FET PET is combined.

## 2 Patients and methods

### 2.1 Patients

We previously evaluated in 62 patients after radiotherapy the diagnostic accuracy of dynamic FET PET for the differentiation of brain metastasis recurrence from radiation injury (Ceccon et al., 2017). In order to perform a PET/MRI textural feature analysis, FET PET data and CE-MRI of these patients were re-evaluated. Of these patients, 52 patients (mean age, 55 ± 10 years (y); range 17–75 y; 39 women and 13 men) were eligible for data evaluation. Ten patients had to be excluded because they were investigated on a different PET scanner.

In brief, these patients with brain metastasis, each having at least one newly diagnosed or progressive contrast-enhancing lesion on cerebral MRI, were referred to our center for the differentiation between brain metastasis recurrence and radiation injury. The median time interval between MRI and PET acquisition was 15 days. Prior to the suspicious CE-MRI, brain metastases had been treated with radiotherapy, i.e., stereotactic radiosurgery, whole-brain radiotherapy, interstitial brachytherapy, external fractionated radiotherapy, or combinations thereof. In detail, forty-five patients (87%) received stereotactic radiosurgery, five patients (10%) had fractionated (local or whole-brain) radiotherapy alone, and two patients (4%) were treated with low-dose brachytherapy. The median time between radiotherapy and suspicious MRI was 15 months (mo; range, 3–64 mo). All patients gave written informed consent before each FET PET investigation. Patients were retrospectively identified and had been seen from 2006 to 2014. The local ethics committee approved the evaluation of retrospectively collected patient data. Patient characteristics are summarized in Table 1.

**Table 1:** Patient and treatment characteristics.

Characteristic		Median	Range	*n* patients
Sex	Woman			39
	Men			13
	Total			52
Age (years) at time of PET imaging		56	17–75	
Primary tumor	Lung (52%)			27
	Breast (29%)			15
	Kidney (6%)			3
	Melanoma (4%)			2
	CUP (2%)			1
	Other^a^ (8%)			4
Type of radiotherapy received before PET	SRS (48%)			27
	SRS and WBRT (37%)			18
	Ext. fract. RT (10%)			4
	Brachytherapy (4%)			2
	WBRT (2%)			1

CUP = cancer of unknown primary; Ext. fract. RT = external fractionated radiotherapy; SRS = stereotactic radiosurgery; WBRT = whole-brain radiotherapy.

a Colorectal carcinoma (n=1); Endometrial carcinoma (n=1); Ewing sarcoma (n=1); Ovarian cancer (n = 1).

The definite diagnosis (brain metastasis recurrence or radiation injury) was based upon histopathology in 19 patients (37%) or follow-up including the clinical course and serial MR imaging in 33 patients (63%). Recurrent disease was anticipated if a new contrast-enhancing lesion appeared at exactly the same site as the previously treated metastasis after initial complete response or if the treated metastasis showed progression in size during follow-up according to Response Assessment in Neuro-Oncology (RANO) criteria for brain metastasis (Lin et al., 2015) (increase of >20% of the pre-treated volume on CE-MRI) and new neurological deficits or the exacerbation of existing neurological symptoms, prompting a change in treatment.

Radiation injuries in the tissue were assumed if (i) the lesions showed spontaneous shrinkage, remained stable in size, or showed a temporally increase of size followed by a spontaneous shrinkage to or below the initial size on CE-MRI during follow-up (median follow-up, 15 mo; range, 3–63 mo); (ii) neurological deficits remained unchanged; (iii) and no new neurological symptoms occurred. In four cases rated as radiation injury, the follow-up time was shorter than 6 months so that stable disease cannot be completely ruled out. In these cases, the classification as radiation injury was additionally based on a negative FET PET scan.

More details about the patient cohort and the clinical follow-up are provided in Supplementary Table 1.

### 2.2 MR Imaging

Standard MR imaging procedures comprised T1-weighted contrast-enhanced axial series, T2-weighted, and fluid attenuated inversion recovery (FLAIR) sequences. As described previously, only the axial T1-weighted contrast-enhanced sequences were used for data evaluation (Pallavi et al., 2014).

### 2.3 FET PET Imaging

The amino acid FET was produced via nucleophilic ^18^F-fluorination with a radiochemical purity >98%, a specific radioactivity >200 GBq/μmol, and a radiochemical yield of about 60%, as previously described (Hamacher and Coenen, 2002). All patients fasted for at least 4 h before the PET measurement according to the German guidelines for brain tumor imaging using labelled amino acid analogues (Langen et al., 2011). All patients underwent a dynamic PET scan from 0 to 50 min post injection (p.i.) of 3 MBq of FET per kg of body weight on a stand-alone standard PET scanner (ECAT EXACT HR+, Siemens Medical Systems, Inc.) in 3D mode (32 rings, axial field of view, 15.5 cm). The reconstructed dynamic dataset consisted of 16 time frames (5 × 1 min; 5 × 3 min; 6 × 5 min). Attenuation correction was based on a 10 min transmission scan measured with three rotating line sources (^68^Ge/^68^Ga). Data were corrected for random and scattered coincidences, and dead time prior to iterative reconstruction using the OSEM algorithm (16 subsets, 6 iterations).

### 2.4 Image pre-processing and VOI definition

**Fig. 1:**
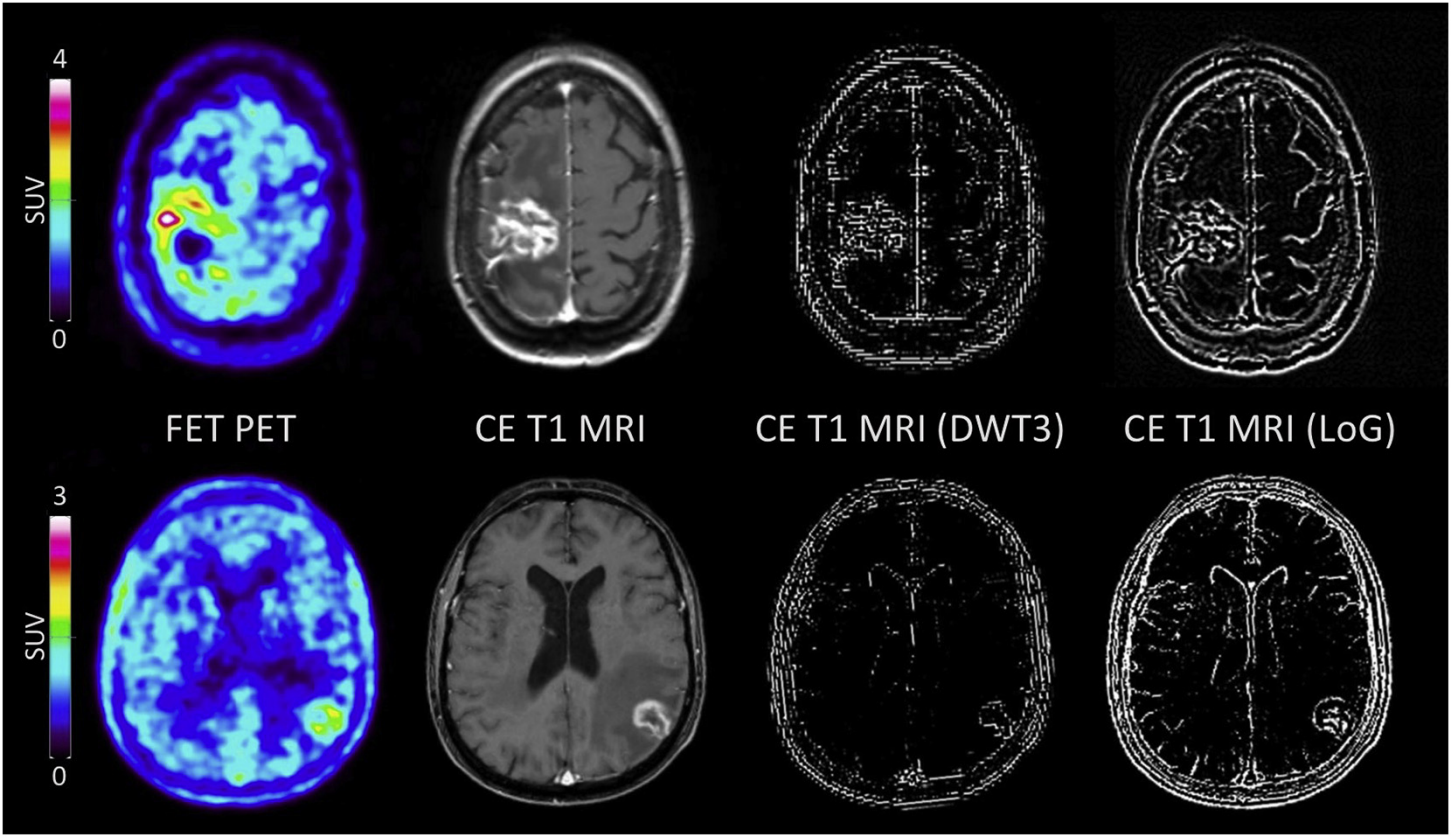
FET PET images, unfiltered and filtered T1-weighted contrast-enhanced (CE) MR images using discrete 3-dimensional wavelet transformation (DWT3) and Laplacian-of-Gaussian (LoG) filtering in a patient (patient #8) with a histologically confirmed recurrent breast cancer metastasis after whole-brain radiotherapy and radiosurgery (upper panel). The lower panel shows a patient (patient #41) who underwent radiosurgery of a brain metastasis originating from a cancer of unknown primary and developed a radiation injury after 21 months of follow-up.

Using the software PMOD (version 3.505, PMOD Technologies Ltd., Zurich, Switzerland), the CE-MR images were resliced to a resolution of 1 × 1 × 1 mm. A B0-field correction was not applied. In patients with multiple lesions, only the lesion with the largest volume was used for textural feature analysis, because this method provides reliable results by selecting regions that contain a sufficient number of voxels (> 100 voxels) (Orlhac et al., 2017a). The Volume-of-Interest (VOI) was defined by the maximal extension of the enhancing region on the T1-weighted contrast-enhanced axial images and was manually contoured in all subsequent slices by an experienced radiation oncologist (M.K.) blinded to the etiology of the lesion (i.e., radiation injury or recurrent metastasis). For further analysis, the CE-MR images were used (i) without additional post-processing, or (ii) after application of a high-pass filter using the Laplacian-of-Gaussian 2-dimensional image filter (LoG) with a sigma of 0.5 mm and a matrix size of 5 × 5 pixels implemented in MATLAB (version R2016b, The MathWorks Inc., Natick, MA, USA), or iii) after application of a high-pass filter using a discrete, first-level 3-dimensional wavelet transformation with the ‘coifl’ wavelet and reconstruction of the higher spatial frequency content in all directions (DWT3). These additional filters enhance the edges of images and are commonly used in textural feature analysis as they make the feature extraction process more sensitive to small-scale changes of tissue properties (Fig. 1) (Kickingereder et al., 2016a; Kickingereder et al., 2016b; Yasaka et al., 2017).

The FET PET VOIs were determined by a 3D auto-contouring process using a TBR of 1.6 or more. This threshold is based on a biopsy-controlled study in which this value separated best between vital tumor and healthy brain parenchyma in FET PET (Pauleit et al., 2005). In cases of non-enhancing PET lesions (*n* = 7), the MR VOIs were registered to the PET images and used for further analysis. No additional filtering was applied for the PET images.

### 2.5 Radiomic feature extraction

Textural feature analysis was performed using the freely available software LIFEx (Version 3.3, lifexsoft.org) (Nioche et al., 2017). Forty-two features were calculated for each VOI including five statistical indices (minimum, mean, maximum, standard deviation of grey levels from the histogram, and VOI volume), 4 first-order histogram features, 31 second-order features from the grey level co-occurrence matrix (GLCM), neighborhood grey-level different matrix (NGLDM), grey-level run length matrix (GLRLM), and grey-level zone length matrix (GLZLM), and two shape indices (sphericity and compacity). A detailed description of each textural parameter is available in the technical appendix of the LIFEx software (Orlhac et al., 2017a). For the second-order features in MRI and FET PET, intensity resampling was performed using the mean and three standard deviations of the grey levels in the VOI as lower and upper limits and rescaling to 64 bins. In 6 cases (Patient number 28, 30, 35, 38, 48, 49), the PET VOIs contained <100 voxels and were therefore excluded from the analysis (Supplementary Table 1) (Orlhac et al., 2017a).

### 2.6 Radiomic feature selection

Calculating large numbers of features on a limited number of patients potentially includes substantial redundancy and might lead to overfitting and misclassification in modelling. Therefore, feature selection for identification of a useful, restricted subset of features for classification of radiation injury from recurrent brain metastasis was performed. First, the Mann-Whitney-*U* test was used to identify features from MRI and FET PET separately as well as from the combination of both modalities that encode statistically significant (*p* < .05) intergroup differences. Second, the maximum number of features allowed in each single modality model and the combined FET PET/MRI model was restricted to five features according to published recommendations (Harrell Jr. et al., 1996; Vittinghoff and McCulloch, 2007).

### 2.7 Model generation

The best performing generalized linear model (logistic regression) was generated using the ‘bestglm’ R-package (Version 3.4.1, R Studio, Inc., Boston, MA, USA) by applying the Akaike Information Criterion (Akaike, 1974). The algorithm was parameterized to select the best model that contained a maximum of five variables according to feature selection. Model generation was applied to the features from the two imaging modalities separately as well as to the combined feature set.

### 2.8 Model validation

The validity of the models for differentiation of recurrent metastasis from radiation injury was assessed using cross-validation (MATLAB, R2017b. Mathworks, Inc., MA, USA). Three commonly used cross-validation methods (leave-one-out, 5-fold, and 10-fold cross-validation) were applied to the models based on the two imaging modalities and the combined model. Additionally, sensitivity, specificity, accuracy, and receiver operating characteristic curves (ROC) were calculated for each modality and the combined feature sets.

## 3 Results

Recurrent metastatic tumor was found in 21 (40%) and radiation injury in 31 (60%) of 52 patients.

**Fig. 2:**
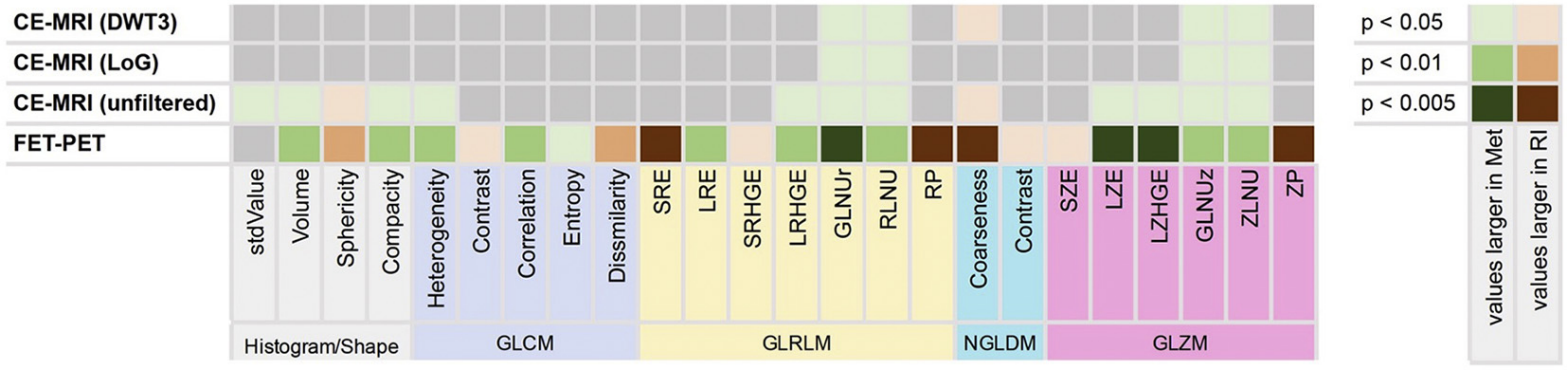
Heat map for textural features with a significant different distribution (two-sided Mann-Whitney-*U*‐test) in patients with recurrent metastasis (Met) compared to those with radiation injury (RI). DWT3: Discrete 3-dimensional wavelet transformation; GLCM: Grey-level co-occurrence matrix; GLNUr: Grey-level non-uniformity for run; GLNUz: Grey-level non-uniformity for zone; GLRLM: Grey-level run-length matrix; GLZLM: Grey-level zone-length matrix; LoG: Laplacian-of-Gaussian filter; LRE: Long-run emphasis; LRHGE: Long-run high grey-level emphasis; LZE: Long-zone emphasis; LZHGE: Long-zone high grey-level emphasis; NGLDM: Neighborhood grey-level different matrix; RLNU: Run length non-uniformity; RP: Run percentage; SRE: Short-run emphasis; SRHGE: Short-run high grey-level emphasis; SZE: Short-zone emphasis; ZLNU: Zone length non-uniformity; ZP: Zone percentage.

Of the 42 analyzed MRI features, 22 features were significantly more frequent in patients with brain metastasis recurrence than in patients with radiation injury (*p* < .05; 13 features from the unfiltered images, 4 features from the LOG filtered and 5 features from the wavelet filtered images) (Fig. 2, Supplementary Tables 2 and 3). The best logistic regression five-variable model yielded a sensitivity of 67%, a specificity of 90%, and an accuracy of 81% (Table 2). The overall accuracy of the model validation was 71% for leave-one-out cross-validation (LOOCV), 77% for 5-fold cross-validation (CV) and 74% for 10-fold cross-validation. Further details on the model performance and the validation are provided in Table 2.

**Table 2:** Summary of best multivariate models and results from model validation.

		FET PET	CE-MRI	Combined
Included features		PET_Volume	T1_stdValue	T1_LZE
		PET_GLNUr	T1_Volume	T1_GLNUz
		PET_RLNU	T1_Compacity	T1_ZLNU
		PET_LZHGE	T1_RLNU	T1_DWT3_GLNUz
		PET_GLNUz	T1_LoG_ZLNU	PET_SRE
Accuracy		83%	81%	89%
Sensitivity		88%	67%	85%
Specificity		75%	90%	96%
AUC		0.91	0.85	0.96
Model validation				
LOOCV	Accuracy	72%	71%	83%
	Sensitivity	77%	81%	85%
	Specificity	65%	57%	80%
	AUC	0.75	0.74	0.86
5-fold CV	Accuracy	74%	77%	80%
	Sensitivity	81%	84%	85%
	Specificity	65%	67%	75%
	AUC	0.76	0.75	0.85
10-fold CV	Accuracy	76%	74%	83%
	Sensitivity	85%	81%	81%
	Specificity	65%	62%	85%
	AUC	0.79	0.77	0.84

AUC: Area under the receiver-operating characteristics curve; CI: Confidence interval; CV: Cross-validation; DWT3: Discrete 3-dimensional wavelet transformation; GLNUr: Grey-level non-uniformity for run; GLNUz: Grey-level non-uniformity for zone; LoG: Laplacian-of-Gaussian filter; LOOCV: Leave-one-out cross-validation; LZE: Long-zone emphasis; LZHGE: Long-zone high grey-level emphasis; RLNU: Run length non-uniformity; ZLNU: Zone length non-uniformity.

Similarly, 23 FET PET textural feature values were significantly more frequent in patients with brain metastasis recurrence than in patients with radiation injury (p < .05 for 23 features, *p* < .01 for 19 features, *p* < .005 for 8 features; Fig. 2 and Supplementary Table 4). The best logistic regression model including 5 variables yielded a sensitivity to detect a recurrence of 88%, a specificity of 75%, and a diagnostic accuracy of 83% (Table 2). The overall accuracy of the model validation was 72% for leave-one-out cross-validation (LOOCV), 74% for 5-fold cross-validation (CV) and 76% for 10-fold cross-validation. Further details on the model performance and the validation are provided in Table 2. Representative MRI and FET PET images are shown in Fig. 1.

For the combined analysis of MRI and FET PET, 22 MRI features and the 8 most significant FET PET features were used for a logistic regression using the best generalized linear model algorithm. The best performing five-variable model included three features from conventional MR images, one feature from the wavelet-transformed MR images and one FET PET feature and resulted in a sensitivity of 85%, a specificity of 96%, and an accuracy of 89% (Fig. 2 and Table 2). The overall accuracy of the model validation was 83% for leave-one-out cross-validation (LOOCV), 80% for 5-fold cross-validation (CV) and 83% for 10-fold cross-validation. Further details on the model performance and the validation are provided in Table 2.

## 4 Discussion

In the present study, we evaluated the ability of both MRI and FET PET radiomic features for the differentiation between brain metastasis recurrence and radiation injury in previously irradiated brain metastases of patients who presented with inconclusive MRI findings. The main finding of our study is that textural features derived from CE-MRI and static FET PET increase the diagnostic accuracy for the correct differentiation of radiation injury from brain metastasis recurrence to almost 90%, compared to each modality alone (82% for CE-MRI, and 83% for FET PET) without the need for a more costly and time-consuming dynamic FET PET acquisition, which is necessary for the evaluation of kinetic PET parameters. Importantly, our analysis was based on standard CE-MRI and static FET PET images that had already been acquired during the routine follow-up of the patients. Thus, no additional measurements or image acquisitions beyond clinical routine were necessary, which speaks for the clinical feasibility of this approach. Although no independent validation cohort was available, the model was validated using cross-validation with different numbers of subsamples, which is a common procedure for model validation with a limited number of samples. Here, the combined model proved valid in the cross-validation yielding high diagnostic accuracies above 80%.

Over the past years, several studies have demonstrated that amino acid PET alone is a potent imaging method for the identification of treatment-related changes such as pseudoprogression (Galldiks et al., 2015; Kebir et al., 2016a; Kebir et al., 2016b; Galldiks, 2017) or radiation injury (Ceccon et al., 2017; Galldiks et al., 2012; Lohmann et al., 2017) in patients with glioma and brain metastasis. For the differentiation of radiation injury from brain metastasis recurrence, the diagnostic accuracy of static (i.e., tumor/brain ratios) and dynamic FET PET parameters (i.e., time-to-peak values and the slope of time-activity curves) has been evaluated in a pilot study by Galldiks et al. (2012). By combining these PET parameters, diagnostic accuracy in the range of 80–90% was obtained. Subsequently, these results have been confirmed by Ceccon et al. in a larger group of patients (Ceccon et al., 2017). Recently, Romagna and colleagues reported a similar diagnostic accuracy (Romagna et al., 2016). However, in all these studies, dynamic FET PET parameters that require a time-consuming (i.e., 40–50 min acquisition time) and hence more expensive PET acquisition, were evaluated. To facilitate data acquisition and analysis, Lohmann and colleagues combined for the first-time textural features derived from static FET PET for the discrimination of radiation injury from recurrent brain metastasis and achieved a diagnostic accuracy of 85% without the acquisition of dynamic FET PET scans (Lohmann et al., 2017).

Although amino acid PET and advanced MRI such as perfusion-weighted imaging or chemical exchange saturation transfer are increasingly gaining attention in Neuro-Oncology (Langen et al., 2017; da Silva et al., 2018), to date conventional MRI is the method of choice in brain tumor diagnostics. Several studies have investigated the potential of conventional MRI for differentiation of radiation injury from brain metastasis recurrence. Using conventional MR imaging, Dequesada et al. (2008) defined a quotient calculated from the solid tumor size in T2-weighted MR images relative to the total extension of contrast enhancement that discriminated patients with recurrent brain metastasis after radiosurgery with high specificity (100%) but low sensitivity (15%). Another approach was used by Kano et al. (2010) who found that a diffuse lesion margin on T2-weighted images compared to a well-delineated margin of contrast enhancement on T1-weighted images (“T1/T2-mismatch”) was significantly associated with a higher rate of radiation injury after radiosurgery (sensitivity, 83%; specificity, 91%). However, these approaches were only qualitative, potentially resulting in a high interobserver variability.

Recently, more advanced MRI-based approaches using quantitative image analysis and machine learning methods have been applied for the differentiation of radiation injury from recurrent brain metastasis. Larozza et al. used a support vector machine classification and extracted 7 predictive features based upon texture analysis resulting in a sensitivity of 83% and a specificity of 82% to detect recurrent metastasis following radiosurgery (Larroza et al., 2015). In another study comprising 25 patients with brain metastasis, when using the five best features Tiwari et al. observed a detection accuracy of 91% in the training set, resulting, however, in a diagnostic accuracy of only 50% in the validation set (Tiwari et al., 2016). A more recent study from Zhang and colleagues used a predictive model after radiosurgery based on MRI features resulting in a diagnostic accuracy of 73% (Zhang et al., 2018).

To the best of our knowledge, this is the first study that combines MR and FET PET radiomics. As CE-MRI and FET PET decode different (patho-)physiological mechanisms that complement each other and may be accessible through textural feature analysis, a combined, multimodal predictor has the potential to outperform single modality models. This assumption is clearly supported by our findings where the combination of both modalities yielded the highest diagnostic performance compared to the single modality models.

As depicted in Fig. 2, many of the textural features that discriminated best between radiation injury and brain metastasis recurrence were found on both MR (unfiltered and filtered) and FET PET images. This is an interesting observation because the underlying mechanisms that determine signal intensities are thought to differ substantially. While the contrast enhancement on MRI represents disruption of the blood-brain barrier (BBB), the increased FET uptake depicted by PET is caused by an overexpression of large neutral amino acid transporters (LAT) leading to an increased accumulation of FET in brain tumors which is not influenced by the BBB permeability (Stegmayr et al., 2017a; Stegmayr et al., 2017b). The observation that the same textural parameters in MRI and FET PET have the highest discriminatory power suggest that patterns of altered amino acid transport and BBB disruption in recurrent metastasis and radionecrosis are altered in the same direction and that there is a fundamental relationship between the physiologically completely different parameters.

Many approaches predominantly in the field of MRI radiomics often use specialized self-developed software that is poorly validated and limits the applicability and accessibility for other groups. Furthermore, high-performance computers are frequently needed for the analysis. In contrast, the software used in the present study is well validated, freely available, and the analysis can be easily performed within a few minutes using routinely acquired multimodal images on a standard computer. In summary, the analysis employed here is readily applicable, easy to implement, and cost-effective.

With regard to the implementation of radiomics into clinical routine, our results warrant further investigation. This should also include a better understanding of the link of specific radiomic features with the underlying pathophysiology, given that it is difficult to translate directly a mathematical description of a radiomic or textural feature into a visual impression or physiological meaning (Orlhac et al., 2017b). In our analysis, patients with brain metastasis recurrence showed a more heterogeneous contrast-enhancement and FET uptake (Fig. 1). Additionally, the shape of the recurrent metastases in both MRI and PET was less spherical compared to radiation injuries (Fig. 1). In the calculated models, particularly features reflecting non-uniformity were dominant and hence achieved higher values in patients with brain metastasis recurrence. Accordingly, findings in recurrent brain metastasis seem to be more heterogeneous than in radiation injuries (Chowdhury et al., 2014; Murrell et al., 2015).

## 5 Conclusions

In conclusion, our results suggest that combined FET PET and MRI radiomics as assessed by textural feature analysis encode more information than either modality alone and is useful for the differentiation between radiation injury and brain metastasis recurrence. Our results warrant both replication and further investigation into the pathophysiology underlying radiomic features.

## Disclosure

This study was partly supported by the Wilhelm Sander-Stiftung, Munich, Germany (No. 2016.069.1 to N.G.). All other authors report no conflicts of interest.

## Appendix A Supplementary data

Supplementary material Image 1
