# Supplementary material for: Combined FET PET/MRI radiomics differentiates radiation injury from recurrent brain metastasis
Source: Neuroimage Clin. 2018 Aug 19;20:537–42. doi: 10.1016/j.nicl.2018.08.024 (PMC6118093; doi:10.1016/j.nicl.2018.08.024)
Supplement: Supplementary file 1 — Supplementary material [file mmc1.doc]

**SUPPLEMENTARY MATERIAL**

**Combined FET PET/MRI radiomics differentiates radiation injury from recurrent brain metastasis**

Neuroimage: Clinical

*****Philipp Lohmann^1,#^, *****Martin Kocher, Garry Ceccon, Elena K. Bauer, Gabriele Stoffels, Shivakumar Viswanathan, Maximilian I. Ruge, Bernd Neumaier, Nadim J. Shah, Gereon R. Fink, Karl-Josef Langen, and Norbert Galldiks

***** both authors have equally contributed to this work

^#^ corresponding author

^1^Inst. of Neuroscience and Medicine (INM-4), Forschungszentrum Juelich, Juelich, Germany

**Supplementary Table 1** Patient cohort and treatment characteristics

| **Pat. #** | **Age [y]** | **Histology of the Primary Tumour** | **Previous Radiotherapy** | **Histologic Diagnosis after PET** | **Follow-up after PET [mo]** | **Final Diagnosis** |
| --- | --- | --- | --- | --- | --- | --- |
| 1 | 45 | DC | WBRT, SRS | Yes | ND | Met |
| 2 | 58 | NSCLC | SRS | Yes | ND | RI |
| 3 | 63 | NSCLC | RT | Yes | ND | RI |
| 4 | 61 | NSCLC | WBRT, SRS | Yes | ND | RI |
| 5 | 45 | DC | WBRT, SRS | Yes | ND | RI |
| 6 | 69 | DC | SRS | Yes | ND | RI |
| 7 | 50 | DC | WBRT, SRS | Yes | ND | Met |
| 8 | 51 | DC | WBRT, SRS | Yes | ND | Met |
| 9 | 17 | Ewing sarcoma | RT | Yes | ND | Met |
| 10 | 57 | NSCLC | SRS | Yes | ND | Met |
| 11 | 50 | MM | SRS | No | 0 | Met |
| 12 | 47 | NSCLC | WBRT, SRS | No | 38 | RI |
| 13 | 69 | NSCLC | SRS | No | 7 | RI |
| 14 | 53 | Colorectal carcinoma | WBRT, SRS | No | 6 | RI |
| 15 | 54 | DC | WBRT, SRS | No | 0 | Met |
| 16 | 58 | NSCLC | SRS | No | 6* | RI |
| 17 | 44 | NSCLC | SRS | No | 16 | RI |
| 18 | 43 | SCLC | WBRT, SRS | No | 16 | RI |
| 19 | 58 | NSCLC | WBRT, SRS | No | 0 | Met |
| 20 | 49 | NSCLC | WBRT, SRS | No | 3* | RI |
| 21 | 58 | DC | SRS | No | 63 | RI |
| 22 | 58 | SCLC | WBRT, SRS | No | 6* | RI |
| 23 | 55 | DC | RT | No | 1 | Met |
| 24 | 70 | DC | RT | No | 0 | Met |
| 25 | 53 | RCC | SRS | No | 3* | RI |
| 26 | 70 | NSCLC | SRS | Yes | ND | Met |
| 27 | 63 | DC | WBRT, SRS | No | 12 | RI |
| 28 | 74 | NSCLC | Brachytherapy | Yes | ND | Met |
| 29 | 34 | DC | WBRT, SRS | Yes | ND | Met |
| 30 | 48 | NSCLC | WBRT | No | 6 | RI |
| 31 | 46 | NSCLC | Brachytherapy | Yes | ND | Met |
| 32 | 58 | Endometrial carcinoma | WBRT, SRS | Yes | ND | Met |
| 33 | 62 | NSCLC | SRS | Yes | ND | Met |
| 34 | 56 | NSCLC | SRS | Yes | ND | Met |
| 35 | 47 | RCC | WBRT, SRS | No | 21 | RI |
| 36 | 46 | DC | SRS | Yes | ND | Met |
| 37 | 66 | DC | SRS | No | 4** | RI |
| 38 | 53 | NSCLC | SRS | No | 3* | RI |
| 39 | 64 | NSCLC | SRS | No | 26 | RI |
| 40 | 56 | NSCLC | SRS | No | 17 | RI |
| 41 | 66 | CUP | SRS | No | 21 | RI |
| 42 | 54 | Ovarian cancer | SRS | No | 13 | RI |
| 43 | 47 | NSCLC | SRS | No | 7 | Met |
| 44 | 58 | NSCLC | WBRT, SRS | Yes | ND | Met |
| 45 | 63 | DC | WBRT, SRS | No | 6 | Met |
| 46 | 52 | DC | SRS | No | 18 | RI |
| 47 | 67 | NSCLC | SRS | No | 7 | RI |
| 48 | 45 | NSCLC | SRS | No | 11 | RI |
| 49 | 37 | MM | SRS | No | 16 | RI |
| 50 | 68 | RCC | SRS | No | 11 | RI |
| 51 | 54 | NSCLC | SRS | No | 20 | RI |
| 52 | 63 | NSCLC | SRS | No | 12 | RI |

**CUP** = Cancer of unknown primary; **DC** = Ductal carcinoma; **Met** = Recurrent brain metastasis; **MM** = Malignant melanoma; **ND** = Not determined; **NSCLC** = Non small-cell lung cancer; **RCC** = Renal cell carcinoma; **RI** = Radiation injury; **RT** = External fractionated radiotherapy; **SCLC** = Small-cell lung cancer; **SRS** = Stereotactic radiosurgery; **WBRT** = Whole-brain radiation therapy; ***** = Lost of follow-up after an observational period of 3 / 6 months; **Patient died after 4 months, cause of death was not related to tumour illness

**Supplementary Table 2** Textural feature values based on unfiltered contrast-enhanced T1 MR images (CE-MRI) significantly differentiating between patients with recurrent metastasis (Met) and radiation injury (RI)

| **Feature** | **Diagnosis** | **n** | **Mean** | **SD** | **Significance** |
| --- | --- | --- | --- | --- | --- |
| **CE-MRI (unfiltered)** | | | | | |
| **Histogram/Shape** |  | | | | |
| T1_stdValue | RI | 31 | 102.05 | 46.91 | p < 0.05 |
|  | Met | 21 | 148.45 | 75.22 |  |
| T1_Volume [mL] | RI | 31 | 5.62 | 5.80 | p < 0.05 |
|  | Met | 21 | 10.14 | 8.74 |  |
| T1_Sphericity | RI | 31 | 0.99 | 0.04 | p < 0.05 |
|  | Met | 21 | 0.97 | 0.03 |  |
| T1_Compacity | RI | 31 | 3.53 | 1.16 | p < 0.05 |
|  | Met | 21 | 4.36 | 1.37 |  |
| **GLCM** |  | | | | |
| T1_Homogeneity | RI | 31 | 0.26 | 0.03 | p < 0.05 |
|  | Met | 21 | 0.28 | 0.04 |  |
| **GLRLM** |  | | | | |
| T1_LRHGE | RI | 31 | 1353.01 | 54.28 | p < 0.05 |
|  | Met | 21 | 1400.88 | 89.72 |  |
| T1_GLNUr | RI | 31 | 145.55 | 148.36 | p < 0.05 |
|  | Met | 21 | 255.84 | 215.53 |  |
| T1_RLNU | RI | 31 | 4753.64 | 4781.30 | p < 0.05 |
|  | Met | 21 | 8265.39 | 6922.36 |  |
| **NGLDM** |  | | | | |
| T1_Coarseness | RI | 31 | 0.005 | 0.005 | p < 0.05 |
|  | Met | 21 | 0.003 | 0.002 |  |
| **GLZLM** |  | | | | |
| T1_LZE | RI | 31 | 16.41 | 14.96 | p < 0.05 |
|  | Met | 21 | 45.73 | 68.29 |  |
| T1_LZHGE | RI | 31 | 15626.63 | 11530.72 | p < 0.05 |
|  | Met | 21 | 34787.40 | 32071.49 |  |
| T1_GLNUz | RI | 31 | 65.83 | 62.49 | p < 0.05 |
|  | Met | 21 | 105.55 | 84.94 |  |
| T1_ZLNU | RI | 31 | 966.32 | 903.77 | p < 0.05 |
|  | Met | 21 | 1492.39 | 1140.16 |  |

**GLCM** = Grey-level co-occurrence matrix; **GLNUr** = Grey-level non-uniformity for run; **GLNUz** = Grey-level non-uniformity for zone; **GLRLM** = Grey-level run-length matrix; **GLZLM** = Grey-level zone-length matrix; **LRHGE** = Long-run high grey-level emphasis; **LZE** = Long-zone emphasis; **LZHGE** = Long-zone high grey-level emphasis; **NGLDM** = Neighbourhood grey-level different matrix; **RLNU** = Run length non-uniformity; **ZLNU** = Zone length non-uniformity

**Supplementary Table 3** Textural feature values based on filtered contrast-enhanced T1 MR images (CE-MRI) significantly differentiating between patients with recurrent metastasis (Met) and radiation injury (RI)

| **Feature** | **Diagnosis** | **n** | **Mean** | **SD** | **Significance** |
| --- | --- | --- | --- | --- | --- |
| **CE-MRI (LoG filter)** | | | | | |
| **GLRLM** |  | | | | |
| LOG_GLNUr | RI | 31 | 157.54 | 162.77 | p < 0.05 |
|  | Met | 21 | 284.51 | 244.48 |  |
| LOG_RLNU | RI | 31 | 4956.86 | 5165.19 | p < 0.05 |
|  | Met | 21 | 8917.08 | 7691.24 |  |
| **GLZLM** |  | | | | |
| LOG_GLNUz | RI | 31 | 81.33 | 84.61 | p < 0.05 |
|  | Met | 21 | 144.15 | 122.55 |  |
| LOG_ZLNU | RI | 31 | 1566.99 | 1668.52 | p < 0.05 |
|  | Met | 21 | 2824.71 | 2411.40 |  |
| **CE-MRI (DWT3 filter)** | | | | | |
| **GLRLM** |  | | | | |
| DWT3_GLNUr | RI | 31 | 167.87 | 172.58 | p < 0.05 |
|  | Met | 21 | 304.06 | 261.18 |  |
| DWT3_RLNU | RI | 31 | 5109.26 | 5278.85 | p < 0.05 |
|  | Met | 21 | 9199.78 | 7919.94 |  |
| **NGLDM** |  | | | | |
| DWT3_Coarseness | RI | 31 | 0.0027 | 0.0028 | p < 0.05 |
|  | Met | 21 | 0.0014 | 0.0013 |  |
| **GLZLM** |  | | | | |
| DWT3_GLNUz | RI | 31 | 95.53 | 94.20 | p < 0.05 |
|  | Met | 21 | 169.81 | 143.87 |  |
| DWT3_ZLNU | RI | 31 | 2036.54 | 2028.23 | p < 0.05 |
|  | Met | 21 | 3659.43 | 3117.74 |  |

**DWT3** = Discrete 3-dimensional wavelet transformation; **GLCM** = Grey-level co-occurrence matrix; **GLNUr** = Grey-level non-uniformity for run; **GLNUz** = Grey-level non-uniformity for zone; **GLRLM =** Grey-level run-length matrix; **GLZLM =** Grey-level zone-length matrix; **LoG** = Laplacian-of-Gaussian filter; **LRHGE** = Long-run high grey-level emphasis; **LZE** = Long-zone emphasis; **LZHGE** = Long-zone high grey-level emphasis; **NGLDM =** Neighbourhood grey-level different matrix; **RLNU** = Run length non-uniformity; **ZLNU** = Zone length non-uniformity

**Supplementary Table 4** Textural feature values based on FET PET images significantly differentiating between patients with recurrent metastasis (Met) and radiation injury (RI)

| **Feature** | **Diagnosis** | **n** | **Mean** | **SD** | **Significance** |
| --- | --- | --- | --- | --- | --- |
| **FET PET** | | | | | |
| **Histogram/Shape** |  | | | | |
| PET_Volume [mL] | RI | 26 | 5.84 | 5.83 | p < 0.01 |
|  | Met | 20 | 13.16 | 10.65 |  |
| PET_Sphericity | RI | 26 | 1.00 | 0.05 | p < 0.01 |
|  | Met | 20 | 0.97 | 0.05 |  |
| PET_Compacity | RI | 26 | 1.76 | 0.61 | p < 0.01 |
|  | Met | 20 | 2.45 | 0.78 |  |
| **GLCM** |  | | | | |
| PET_Homogeneity | RI | 26 | 0.20 | 0.02 | p < 0.005 |
|  | Met | 20 | 0.23 | 0.04 |  |
| PET_Contrast | RI | 26 | 127.07 | 41.48 | p < 0.05 |
|  | Met | 20 | 94.93 | 33.77 |  |
| PET_Correlation | RI | 26 | 0.47 | 0.16 | p < 0.01 |
|  | Met | 20 | 0.60 | 0.12 |  |
|  | | | | | |
| PET_Entropy | RI | 26 | 2.54 | 0.28 | p < 0.05 |
|  | Met | 20 | 2.73 | 0.19 |  |
| PET_Dissimilarity | RI | 26 | 8.75 | 1.52 | p < 0.01 |
|  | Met | 20 | 7.37 | 1.40 |  |
| **GLRLM** |  | | | | |
| PET_SRE | RI | 26 | 0.98 | 0.01 | p < 0.005 |
|  | Met | 20 | 0.97 | 0.01 |  |
| PET_LRE | RI | 26 | 1.09 | 0.03 | p < 0.01 |
|  | Met | 20 | 1.14 | 0.06 |  |
| PET_SRHGE | RI | 26 | 1109.74 | 10.56 | p < 0.05 |
|  | Met | 20 | 1097.55 | 15.32 |  |
| PET_LRHGE | RI | 26 | 1242.05 | 36.05 | p < 0.01 |
|  | Met | 20 | 1298.90 | 68.66 |  |
| PET_GLNUr | RI | 26 | 18.62 | 17.46 | p < 0.005 |
|  | Met | 20 | 49.96 | 41.79 |  |
| PET_RLNU | RI | 26 | 543.96 | 538.41 | p < 0.01 |
|  | Met | 20 | 1171.35 | 916.39 |  |
|  | | | | | |
| PET_RP | RI | 26 | 0.97 | 0.01 | p < 0.005 |
|  | Met | 20 | 0.96 | 0.02 |  |
| **NGLDM** |  | | | | |
| PET_Coarseness | RI | 26 | 0.02 | 0.01 | p < 0.005 |
|  | Met | 20 | 0.01 | 0.01 |  |
| PET_Contrast | RI | 26 | 0.62 | 0.23 | p < 0.05 |
|  | Met | 20 | 0.49 | 0.12 |  |
| **GLZLM** |  | | | | |
| PET_SZE | RI | 26 | 0.77 | 0.05 | p < 0.01 |
|  | Met | 20 | 0.718 | 0.048 |  |
| PET_LZE | RI | 26 | 3.34 | 1.33 | p < 0.005 |
|  | Met | 20 | 9.48 | 12.66 |  |
| PET_LZHGE | RI | 26 | 3799.31 | 1509.95 | p < 0.005 |
|  | Met | 20 | 9024.40 | 9541.30 |  |
| PET_GLNUz | RI | 26 | 12.23 | 11.41 | p < 0.01 |
|  | Met | 20 | 24.84 | 18.27 |  |
| PET_ZLNU | RI | 26 | 204.23 | 192.58 | p < 0.01 |
|  | Met | 20 | 337.53 | 221.10 |  |
| PET_ZP | RI | 26 | 0.68 | 0.078 | p < 0.005 |
|  | Met | 20 | 0.58 | 0.10 |  |

**GLCM** = Grey-level co-occurrence matrix; **GLNUr** = Grey-level non-uniformity for run; **GLNUz** = Grey-level non-uniformity for zone; **GLRLM =** Grey-level run-length matrix; **GLZLM =** Grey-level zone-length matrix; **LRE** = Long-run emphasis; **LRHGE** = Long-run high grey-level emphasis; **LZE** = Long-zone emphasis; **LZHGE** = Long-zone high grey-level emphasis; **NGLDM =** Neighbourhood grey-level different matrix; **RLNU** = Run length non-uniformity; **RP** = Run percentage; **SRE** = Short-run emphasis; **SRHGE** = Short-run high grey-level emphasis; **SZE** = Short-zone emphasis; **ZLNU** = Zone length non-uniformity; **ZP** = Zone percentage
